# Hepatic stellate cells promote upregulation of epithelial cell adhesion molecule and epithelial-mesenchymal transition in hepatic cancer cells

**DOI:** 10.3892/or.2015.4126

**Published:** 2015-07-13

**Authors:** TERUYA NAGAHARA, HIDENORI SHIRAHA, HIROAKI SAWAHARA, DAISUKE UCHIDA, YASUTO TAKEUCHI, MASAYA IWAMURO, JUNRO KATAOKA, SHIGERU HORIGUCHI, TAKESHI KUWAKI, HIDEKI ONISHI, SHINICHIRO NAKAMURA, AKINOBU TAKAKI, KAZUHIRO NOUSO, KAZUHIDE YAMAMOTO

**Affiliations:** Department of Gastroenterology and Hepatology, Okayama University Graduate School of Medicine, Shikata-cho, Kita-ku, Okayama 700-8558, Japan

**Keywords:** hepatocellular carcinoma, hepatic stellate cell, niche, Notch, EpCAM, cancer stem cells

## Abstract

Microenvironment plays an important role in epithelial-mesenchymal transition (EMT) and stemness of cells in hepatocellular carcinoma (HCC). Epithelial cell adhesion molecule (EpCAM) is known as a tumor stemness marker of HCC. To investigate the relationship between microenvi-ronment and stemness, we performed an *in vitro* co-culture assay. Four HCC cell lines (HepG2, Hep3B, HuH-7 and PLC/PRF/5) were co-cultured with the TWNT-1 immortalized hepatic stellate cells (HSCs), which create a microenvironment with HCC. Cell proliferation ability was analyzed by flow cytometry (FCM) and 3-(4,5-dimethylthiazol-2-yl)-2,5-diphe-nyltetrazolium bromide (MTT) assay, while migration ability was assessed by a wound healing assay. Expression of EpCAM was analyzed by immunoblotting and FCM. HCC cell lines were co-cultured with TWNT-1 treated with small interfering RNA (siRNA) for TGF-β and HB-EGF; we then analyzed proliferation, migration ability and protein expression using the methods described above. Proliferation ability was unchanged in HCC cell lines co-cultured with TWNT-1. Migration ability was increased in HCC cell lines (HepG2, Hep3B, HuH-7 and PLC/PRF/5) directly (216.2±67.0, 61.0±22.0, 124.0±66.2 and 51.5±40.3%) and indirectly (102.5±22.0, 84.6±30.9, 86.1±25.7 and 73.9±29.7%) co-cultured with TWNT-1 compared with the HCC uni-culture. Immunoblot analysis revealed increased EpCAM expression in the HCC cell lines co-cultured with TWNT-1. Flow cytometry revealed that the population of E-cadherin^−^/N-cadherin^+^ and EpCAM-positive cells increased and accordingly, EMT and stemness in the HCC cell line were activated. These results were similar in the directly and indirectly co-cultured samples, indicating that humoral factors were at play. Conversely, HCC cell lines co-cultured with siRNA-treated TWNT-1 showed decreased migration ability, a decreased population of EpCAM-positive and E-cadherin^−^/N-cadherin^+^ cells. Taken together, humoral factors secreted from TWNT-1 promote upregulation of EpCAM and EMT in hepatic cancer cells.

## Introduction

Hepatocellular carcinoma (HCC) is the third most frequent cause of cancer-related death worldwide (1,2). There are several treatment modalities for HCC, including surgical resection, radiofrequency ablation, transcatheter arterial chemoembolization and percutaneous ethanol injection therapy. Recent advances in these treatment modalities have improved the prognosis of HCC, yet prognosis for advanced HCC remains poor (1). Several studies investigated the factors associated with refractory HCC have shown that CD133 and epithelial cell adhesion molecule (EpCAM) are related to the poor prognosis (3–5).

Stemness is an essential characteristic of stem cells to renew themselves and differentiate with multipotency. EpCAM is a cell surface marker expressed on human hepatic stem cells and is a known marker to identify cancer stem cells (CSCs) in HCC (6). EpCAM is related to tumorigenesis and metastasis, and its expression is a prognostic factor for HCC (3–5). EpCAM is a downstream effector of the Notch signaling cascade. Activation of Notch signaling enhances cellular stemness and epithelial-mesenchymal transition (EMT) by inducing EpCAM expression in both pancreatic cancer (7) and HCC (8). Moreover, other investigators have reported that EpCAM plays an important role in EMT (9,10).

Cancer progression is related to genetic changes within cancer cells as well as in the microenvironment. Stromal cells in the cancer microenvironment facilitate the development of cancer EMT (11). Cancer cells that undergo EMT also acquire a stem cell-like phenotype (12), suggesting that cancer cell-microenvironment interactions are important in cancer progression. In various types of cancers, such as intrahepatic cholangiocarcinoma (13), oligodendroglioma (14), pancreatic cancer (15), *in vitro* co-culture assays of cancer cell lines and cells in the cancer microenvironment increases EMT. In the present study, we hypothesized that the microenvironment associated with HCC enhances EMT. Hepatic stellate cells (HSCs) are liver-specific mesenchymal cells located in perisinusoidal and portal areas. HSCs play an important role in the stem cell niche for hepatic progenitor cells and hepatocytes. In addition, HSCs are known to present histopathologically among HCC tissue (16), and are thought to make a niche for hepatic cancer cells. Therefore, in the present study, we investigated the interaction between HSCs and HCC cells.

## Materials and methods

### Cell lines and culture

The human HCC cell lines HepG2, Hep3B, HuH-7 and PLC/PRF/5 were obtained from American Type Culture Collection (ATCC; Manassas, VA, USA). Immortalized human HSC cells (TWNT-1) were a generous gift from Dr Naoya Kobayashi from the Department of Gastroenterological Surgery, Okayama University School of Medicine. Cells were maintained in high glucose Dulbecco's modified Eagle's medium (DMEM; Invitrogen, Carlsbad, CA, USA) supplemented with 10% heat-inactivated fetal bovine serum (FBS), 1% non-essential amino acids, penicillin/streptomycin solution (both from Sigma-Aldrich, St. Louis, MO, USA). Cells were cultured at 37°C in an atmosphere of 5% CO_2_ and 95% air. The cells were treated under restricted serum conditions with 0.5% dialyzed FBS for 24 h before the experiment when necessary.

### Direct co-culture of hepatic cancer cells and HSCs

HCC cell lines [400,000 cells/well (HepG2), 200,000 cells/well (Hep3B, HuH-7 and PLC/PRF/5)] and TWNT-1 (50,000 cells/well) were seeded in 6-well culture plates (353046; Corning, Corning, NY, USA) in DMEM supplemented with 0.5% dialyzed FBS and 1% supplements as previously described, and incubated for 3 days. If required, HSCs were pre-treated with mitomycin C before they were used for assays in order to inhibit self-proliferation. After this, cells were seeded and cultured in this manner in case of direct co-culture unless otherwise specified.

### Indirect co-culture of hepatic cancer cells and HSCs

HCC cell lines [400,000 cells/well (HepG2), 200,000 cells/well (Hep3B, HuH-7 and PLC/PRF/5)] were seeded in 6-well culture plates in DMEM supplemented with 0.5% dialyzed FBS and 1% supplements as previously described. TWNT-1 (50,000 cells/well) were seeded into the Cell Culture Insert™ of 1.0-*µ*m pore size (353102; Corning) in the same medium as used for HCC cells, inserted into the 6-well plates where HCC cells were seeded, and incubated for 3 days. After this, cells were seeded and cultured in this manner in case of indirect co-culture unless otherwise specified.

### Immunoblot analysis

HCC cells were seeded in 6-well culture plates and uni-cultured and indirectly co-cultured with TWNT-1, and grown to confluence. The HCC cells were washed twice with cold Dulbecco's phosphate-buffered saline (DPBS) and lysed in 100 *µ*l of sample buffer [100 mM Tris-HCl (pH 6.8), 10% glycerol, 4% sodium dodecyl sulfate (SDS), 1% bromophenol blue and 10% β-mercaptoethanol]. The samples were resolved by SDS-polyacrylamide gel electrophoresis and transferred to a polyvinylidene difluoride (PVDF) membrane (Bio-Rad, Hercules, CA, USA). The membranes were blocked using PVDF blocking reagent Can Get Signal™ (Toyobo, Osaka, Japan) for 1 h. The membranes were then incubated with antibodies against cleaved Notch [#2421; Cell Signaling Technology (CST), Danvers, MA, USA], Hes1 (ab49170; Abcam, Cambridge, UK), Twist1 [sc-6070; Santa Cruz Biotechnology (SCBT), Dallas, TX, USA], E-cadherin (#3195), N-cadherin (#13116), EpCAM (#2929) and β-actin (#4967S) (all from CST) for 1 h at room temperature. The membranes were washed 3 times with TBS-T and probed with horseradish peroxidase-conjugated secondary antibody (#7074S; CST) before being developed with an ECL blotting detection system (Amersham Biosciences, Piscataway, NJ, USA) using enhanced chemiluminescence.

### Gene silencing of TGF-β and HB-EGF with small interfering RNA (siRNA)

TWNT-1 was transfected with scrambled negative control siRNA and TGF-β1/2/3, and HB-EGF siRNA, respectively (sc-37007, sc-44146 and sc-39420; SCBT). siRNAs were transfected into cells using a siRNA transfection reagent and siRNA transfection medium (sc-45064; SCBT). Cells were seeded at 200,000 cells/well in 6-well culture plates and incubated for 24 h before the 5 h transfection with scrambled negative control, TGF-β1/2/3 and HB-EGF siRNA, and then used for assays after additional incubation for 48 h.

### MTT assay

Cell proliferation ability was evaluated with the 3-(4,5-dimethylthiazol-2-yl)-2,5-diphenyltetrazolium bromide (MTT) assay. HCC cell lines were seeded at 40,000 cells/well in 500 *µ*l of DMEM supplemented with 1% dialyzed FBS in 24-well culture plates (353047; Becton-Dickinson, Franklin Lakes, NJ, USA). TWNT-1 (30,000 cells/well) were seeded into the Cell Culture Insert™ of 1.0-*µ*m pore size (353104; Corning) in the same medium as used for HCC cells, and plated into the 24-well plates where the HCC cells were seeded. After 48 h of quiescence, 50 *µ*l of MTT (5 mg/ml in PBS) was added to each well, and the wells were incubated for an additional 3.5 h at 37°C. The purple-blue MTT formazan precipitate was dissolved in 500 *µ*l of dimethylsulfoxide (Sigma-Aldrich) and applied in 100 *µ*l volumes in a 96-well culture plate in the fourth replicate. The activity of mitochondria, used as a measure of cellular growth and viability, was evaluated by measuring optical density at 570 nm with a microplate reader (Model 680 microplate reader; Bio-Rad).

### Migration assay

Cell migration ability was evaluated by an *in vitro* wound healing assay. HCC cell lines (HepG2, Hep3B, HuH-7 and PLC/PRF/5) were seeded at 500,000, 600,000, 200,000 and 600,000 cells/well, respectively, in 6-well culture plates then uni-cultured, directly and indirectly co-cultured with TWNT-1 (50,000/well) in DMEM supplemented with 10% FBS. TWNT-1 was pre-treated with mitomycin C before use in the direct co-culture assays to inhibit self-proliferation. After cells grew to confluence, the cell monolayer was mechanically scratched with a sterile 200 *µ*l pipette tip and incubation was continued for an additional 48 h in DMEM medium supplemented with 0.5% dialyzed FBS. Images were captured at 0, 24 and 48 h, and the distance traveled by the cells at the acellular front was measured in 3 places.

### Flow cytometric analysis

Cells were seeded in 6-well culture plates and uni-cultured, directly or indirectly co-cultured with HSCs in DMEM medium supplemented with 0.5% dialyzed FBS for 3 days. They were subsequently washed twice with DPBS, collected and re-suspended in 100 *µ*l of S-PBS (DPBS supplemented with 2% FBS and 10 mM HEPES buffer saline solution). Vio-blue-anti-CD44, PE-vio770-anti-CD133, APC-anti-EpCAM, APC-vio770-anti-E-cadherin (130-104-270, 130-104-155, 130-091-254 and 130-101-095; Miltenyi Biotechnology, Bergisch Gladbach, Germany), FITC-anti-CD90 (IM1839U; Beckman Coulter, Brea, CA, USA), and PE-anti-N-cadherin antibodies (350806; BioLegend, San Diego, CA, USA) were added, and cells were incubated on ice for 15 min in the dark. After washing once, cells were re-suspended in 500 *µ*l of S-PBS supplemented with 5 *µ*l of propidium iodide (PI) solution (P378; Dojindo, Kumamoto, Japan) and analyzed using a flow cytometer (MACSQuant, Miltenyi Biotechnology).

### Statistical analysis

The results are expressed as mean ± SD. Experiments were performed at least 3 times. Differences between the groups were evaluated by the Student's t-test. A p-value <0.05 was considered to indicate a statistically significant result.

## Results

### Direct or indirect co-culture with HSCs does not increase proliferation in HCC cell lines

**Figure 1:**
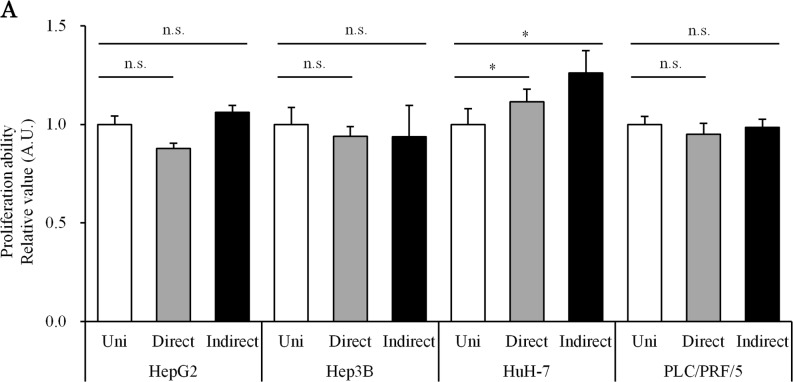

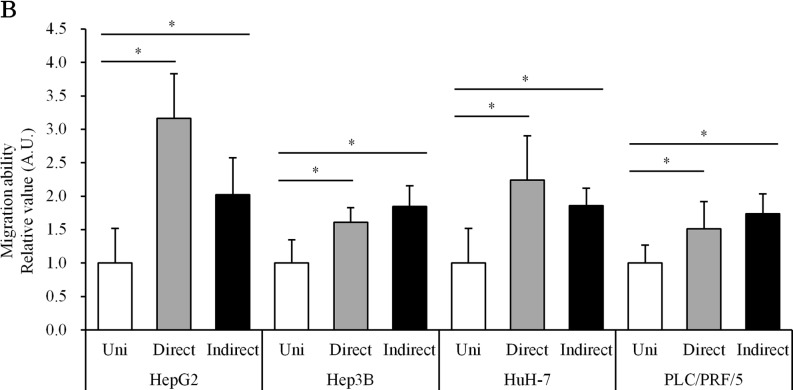
Biological effects in HCC cell lines under co-culture with HSCs. (A) HCC cell lines were directly and indirectly co-cultured with HSCs. Indirect co-culture was performed using a Cell Culture Insert™. Cell proliferative abilities in HCC cell lines were evaluated by flow cytometry. (B) Cell migration activity was measured as described in Materials and methods. Data represent the mean ± SD of >3 independent experiments, each performed in triplicate. n.s., p>0.05; ^*^p<0.05 (vs., data for uni-culture); Student's t-test. HCC, hepatocellular carcinoma; HSCs, hepatic stellate cells.

To assess the biological effect of co-culture with HSCs, the proliferative activities of HCC cell lines were assessed by flow cytometry. This technique allowed us to evaluate the number of viable HCC cells in the mixture. The proliferation ability of the co-culture group was not significantly different from that of the uni-culture group (Fig. 1A).

### Direct and indirect co-culture with HSCs promotes migration in HCC cell lines

We evaluated the migration ability of the uni- and co-culture groups using an *in vitro* wound healing assay. The migration activity under co-culture conditions was higher than that under uni-culture condition in all four HCC cell lines (Fig. 1B). This effect was observed in both direct and indirect co-culture conditions (Fig. 1B).

### Indirect co-culture with HSCs upregulates Notch signaling in HCC cell lines

**Figure 2:**
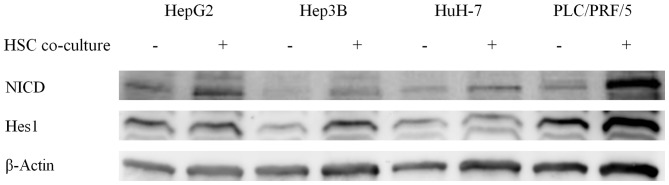
The effect of HSC co-culture on Notch signaling in HCC cell lines. HCC cell lines were co-cultured with HSC using a cell culture insert. After removing HSC cells, lysates were prepared from HCC cells. Immunoblot analyses were performed using antibodies against NICD and Hes1. Immunoblotting for β-actin levels was used to verify equal loading of cellular proteins. Representative blots of >3 independent experiments are shown. HSC, hepatic stellate cell; HCC, hepatocellular carcinoma.

As cell migration ability was increased by co-culture with HSCs, we hypothesized that the co-culture condition enhanced EMT in HCC cell lines. The Notch signaling pathway exists upstream of EMT and regulates it. Therefore, we evaluated the expression of proteins in the Notch signaling pathway by immunoblotting. Notch intracellular domain (NICD) and Hes1 expression were increased in the co-culture group, indicating activation of the Notch signaling pathway (Fig. 2).

### Indirect co-culture with HSCs promotes EMT in HCC cell lines

**Figure 3:**
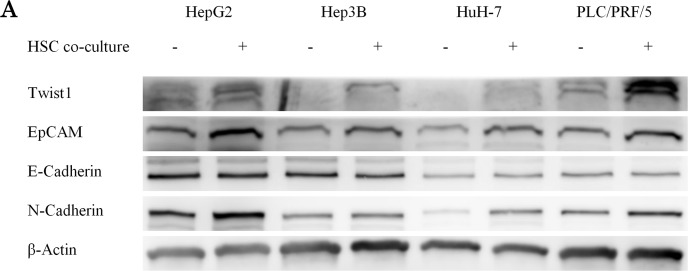

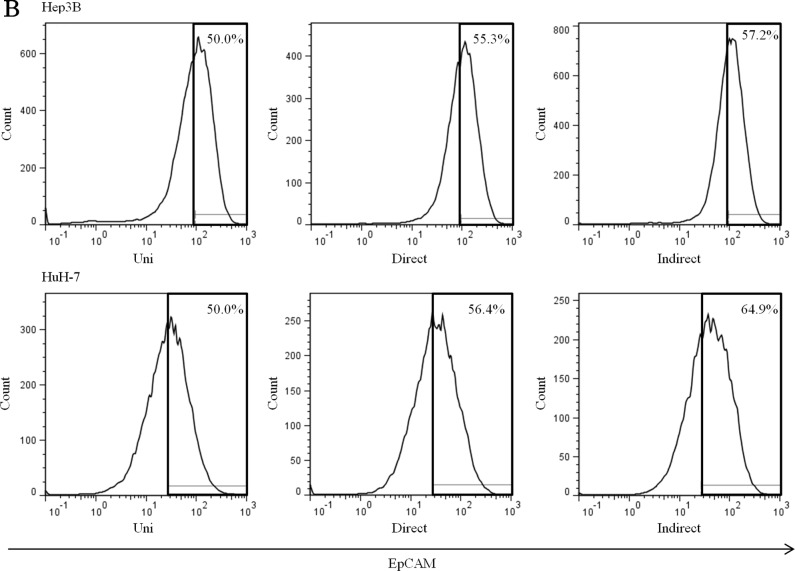

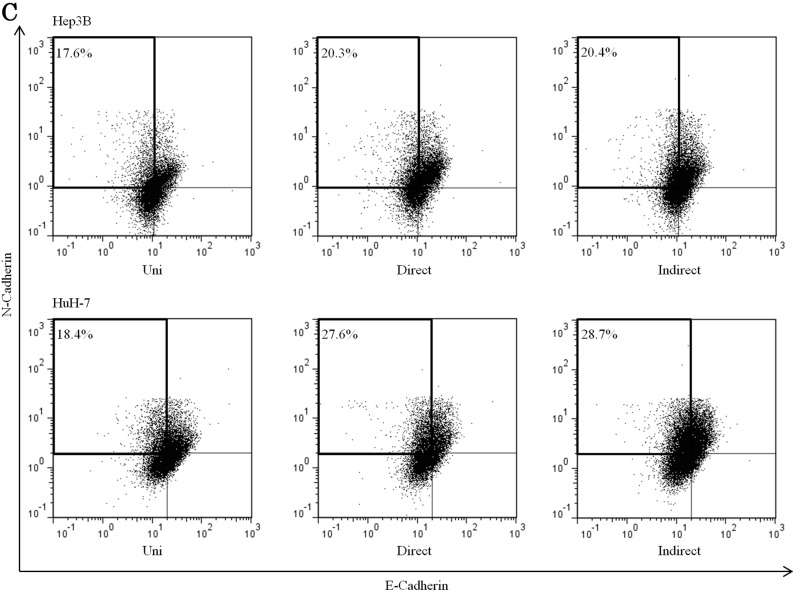
The effect of HSC co-culture on EMT in HCC cell lines. HCC cell lines were co-cultured with HSC using a Cell Culture Insert™. (A) After 72 h co-culture, HCC cells were lysed and used for immunoblot analysis. Immunoblot analyses were performed using antibodies against Twist1, E-cadherin, N-cadherin and EpCAM. Immunoblotting for β-actin was used to verify equal loading of cellular proteins. Representative blots of >3 independent experiments are shown. (B) HCC cells were collected and washed in PBS without Ca^2+^/Mg^2+^ and incubated with APC-conjugated anti-EpCAM antibody. The cells were analyzed with MACSQuant flow cytometer. The representative plots from >3 independent experiments are shown. (C) HCC cells were collected and washed in PBS without Ca^2+^/Mg^2+^ and incubated with APC-vio770-conjugated anti-E-cadherin and PE-conjugated anti-N-cadherin. The cells were analyzed with the flow cytometer. The representative plots from >3 independent experiments are shown. HSC, hepatic stellate cell; EMT, epithelial-mesenchymal transition; HCC, hepatocellular carcinoma; EpCAM, epithelial cell adhesion molecule.

It is known that the expression of EpCAM and Twist are increased in EMT (17,18). We evaluated the expression of the EMT markers Twist1, E-cadherin, N-cadherin and EpCAM by immunoblotting in the uni- and co-culture groups. Both EpCAM and Twist1 expression were increased in the co-culture group (Fig. 3A).

### Direct and indirect co-culture with HSCs increases the proportion of EpCAM-positive HCC cells

We evaluated the expression of E-cadherin and N-cadherin, both of which are associated with EMT, by immunoblotting in the uni- and co-culture groups. The co-culture reduced E-cadherin expression in HepG2 and Hep3B, and induced N-cadherin expression in HepG2, Hep3B, HuH-7 and PLC/PRF/5. As we suspected that only a portion of the cells changed their EMT-marker expression, we evaluated the expressions of EpCAM, E-cadherin and N-cadherin by flow cytometry in both groups. In the co-culture group, we found that the ratio of EpCAM-positive cells increased (Fig. 3B). Furthermore, the percentage of low E-cadherin and high N-cadherin cells increased, suggesting the progression of EMT in the co-culture group (Fig. 3C).

### Suppression of TGF-β and HB-EGF inhibits migration, Notch signaling

Enhanced migration activity and EMT progression were observed in both direct and indirect co-culture groups, changes that we suspected were caused by liquid factors. With the aim of reducing the liquid factors secreted from HSCs, such as TGF-β and HB-EGF, we treated HSCs with siRNA against these two molecules.

**Figure 4:**
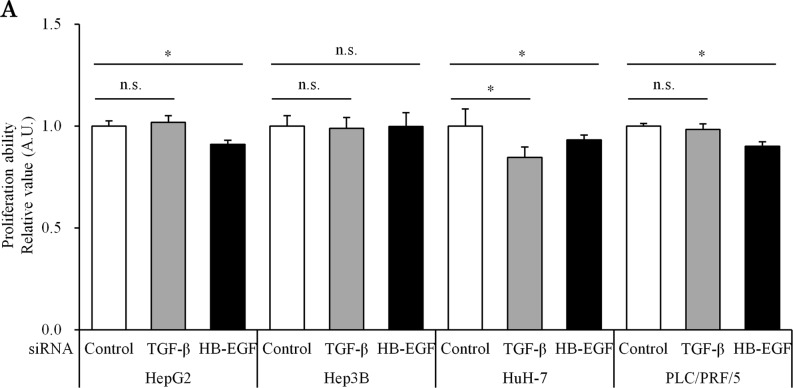

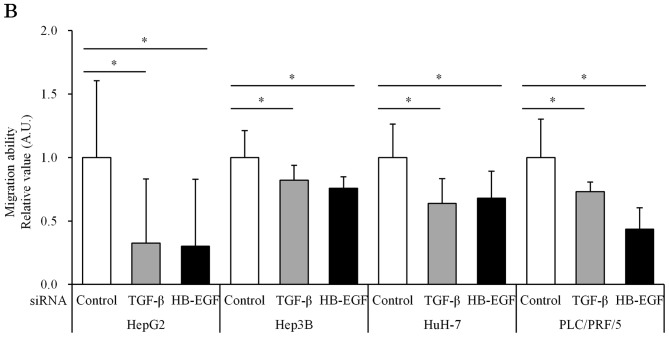

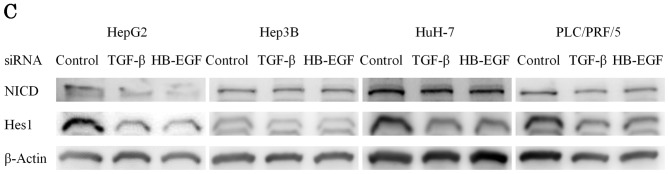
Effect of TGF-β and HB-EGF gene silencing on HCC cell lines co-cultured with HSCs. HSCs were treated with TGF-β1/2/3 and HB-EGF siRNA for 5 h. Then, HCC cell lines were co-cultured with siRNA-treated HSCs for 48 h. (A) Cell proliferation activity was evaluated by MTT assay. Data represent the mean ± SD of >3 independent experiments, each performed in triplicate. n.s., p>0.05; ^*^p<0.05 (vs. data for control siRNA treated group); Student's t-test. (B) Cell migration activity was assessed by an *in vitro* wound healing assay. Data represent the mean ± SD of >3 independent experiments, each performed in triplicate. n.s., p>0.05; ^*^p<0.05 (vs. data for control siRNA treated group); Student's t-test. (C) HCC cells were isolated and lysed. Immunoblot analysis was performed using antibodies against NICD and Hes1. Immunoblotting for β-actin was used to verify equal loading of cellular proteins. Representative blots of >3 independent experiments are shown. HCC, hepatocellular carcinoma; HSCs, hepatic stellate cells.

The siRNA against TGF-β and HB-EGF successfully suppressed TGF-β (51.7±20.2% inhibition) and HB-EGF (69.2±12.3% inhibition) expression in HSC cell lines, respectively. Both TGF-β and HB-EGF siRNA had a minimal effect on cell proliferative activity in HCC cell lines co-cultured with HSCs (Fig. 4A). However, both TGF-β and HB-EGF siRNA reduced the cell migration potential of HCC cell lines under co-culture with HSCs (Fig. 4B).

The expression of proteins in the Notch signaling pathway was analyzed by immunoblotting. Migration activity, and expression of proteins associated with the Notch signaling pathway were decreased in HCC cells treated with siRNA against TGF-β and HB-EGF compared to the control (Fig. 4C).

### Suppression of TGF-β and HB-EGF reduces EpCAM-positive HCC cells and inhibits EMT

**Figure 5:**
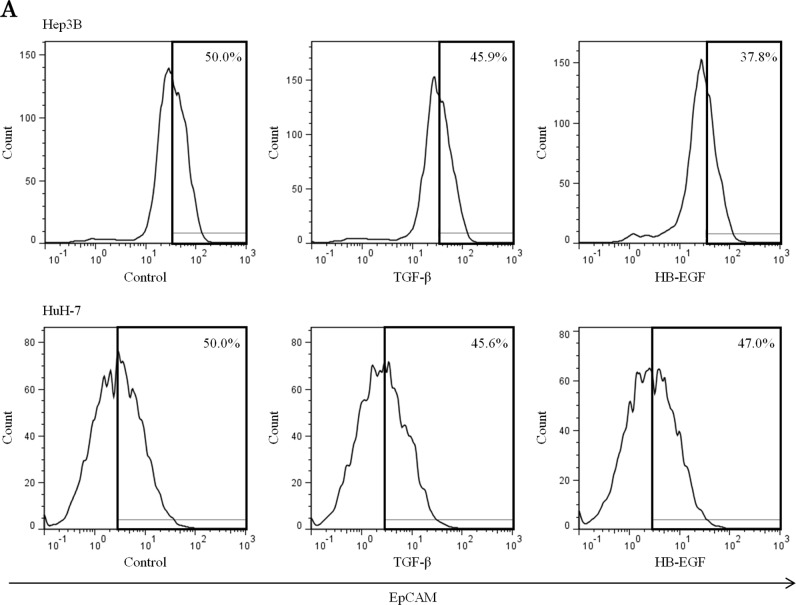

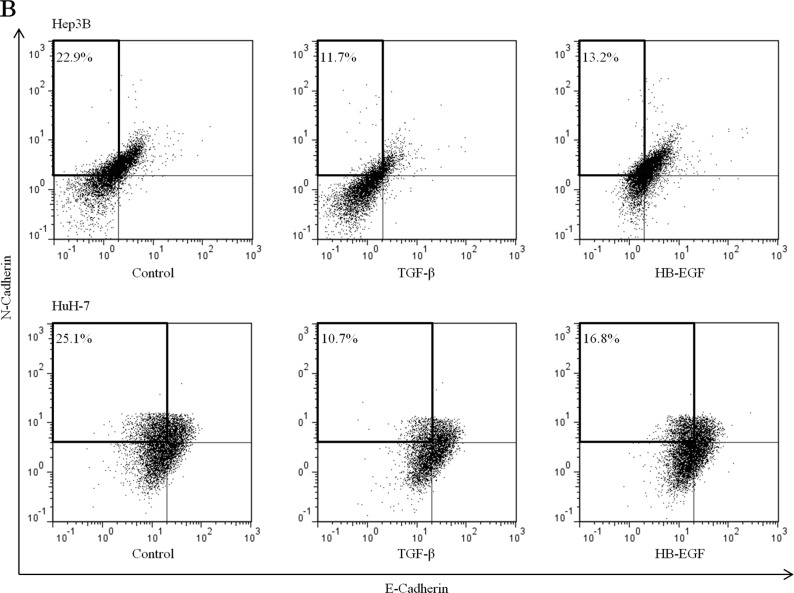
Effect on EMT and stemness of TGF-β and HB-EGF gene silencing in HCC cell lines co-cultured with HSCs. (A) HSCs were treated with TGF-β1/2/3 and HB-EGF siRNA for 5 h. Then, HCC cell lines were co-cultured with siRNA-treated HSCs for 72 h. HCC cells were collected and washed in PBS without Ca^2+^/Mg^2+^ and incubated with APC-conjugated anti-EpCAM antibody. The cells were analyzed with MACSQuant flow cytometer. Representative plots from >3 independent experiments are shown. (B) HSCs were treated with TGF-β1/2/3 and HB-EGF siRNA for 5 h. Then, HCC cell lines were co-cultured with siRNA-treated HSCs for 72 h. HCC cells were collected and washed in PBS without Ca^2+^/Mg^2+^ and incubated with APC-vio770-conjugated anti-E-cadherin and PE-conjugated anti-N-cadherin. The cells were analyzed with the flow cytometer. Representative plots from >3 independent experiments are shown. EMT, epithelial-mesenchymal transition; HCC, hepatocellular carcinoma; HSCs, hepatic stellate cells; EpCAM, epithelial cell adhesion molecule.

We performed flow cytometry to evaluate EMT capability and stemness of HCC cell lines co-cultured with HSCs treated with siRNA against TGF-β and HB-EGF. Both TGF-β and HB-EGF siRNA decreased the population of EpCAM-positive HCC cells (Fig. 5A). The population of HCC cells expressing N-cadherin but lacking E-cadherin occurring in EMT was decreased (Fig. 5B).

## Discussion

Cancerous tumors consist of heterogeneous cancer and stromal cells. The stromal cell-associated cancer microenvironment is known to play critical roles in cancer growth and progression (11,19). In the present study, we demonstrated that HSCs induce EMT in HCC cells. HSCs were chosen among various hepatic stromal cells as they secrete various growth factors and directly affect hepatocytes (20). The immunohistochemical analyses revealed that HSCs were present in the stroma of HCC (16). Furthermore, the activation of HSCs in the medium promoted tumorigenicity of HCC (21). It is also reported that activated peritumoral HSCs are connected to high and early tumor recurrence, and increased death rate (22). Collectively, the evidence suggests that HSCs contribute to HCC progression.

We first assessed the biological impact on HCC cells under co-culture conditions with HSC (Fig. 1). We observed that the co-culture did not affect the proliferation of HCC cells (Fig. 1A), while the migration ability of HCC cells was significantly enhanced (Fig. 1B). This effect was observed in both direct and indirect co-culture experiments, suggesting that this effect was induced by soluble factors.

It is reported that glioblastoma and cholangiocarcinoma cells with their respective astrocytes enhance EMT and stemness in cancer cells (13,14). Pancreatic cancer cell lines are also reported to enhance EMT when cultured with pancreatic stellate cells (15). The growing body of evidence indicates that the stromal microenvironment contributes to cancer progression. In accordance with these studies, the HSC-related microenvironment induced EMT in HCC cells in the present study.

EMT plays an important role in cancer progression as well as normal embryogenesis (23). The cancer microenvironment contributes to cancer progression and induces the occurrence of EMT in cancer cells (11,24). As the migration ability is closely associated with EMT, we conducted an immunoblot analysis for EMT-related molecules (Figs. 2 and 3A). The co-culture condition decreased the expression of E-cadherin in HCC cells and increased the expression of N-cadherin. Notably, the co-culture condition also induced the expression of Twist, a strong EMT inducer and EpCAM in HCC cells (Fig. 3A).

Our previous study demonstrated that the expression of EpCAM is suppressed by the expression of runt-related transcription factor 3 (RUNX3) and that the Notch signaling pathway is reduced by RUNX3 expression (8). This evidence suggests that EpCAM expression and Notch signaling is closely related. We subsequently assessed the effect of co-culture on Notch signaling (Fig. 2). Co-culture conditions activated a downstream signaling molecule of Notch in HCC cells, as expected (Fig. 2).

In order to analyze the population of EMT-induced cells, flow cytometric analysis was conducted, which revealed that the co-culture increased the ratio of EpCAM-positive cells in Hep3B and HuH-7 (Fig. 3B). The population of cells with low expression of E-cadherin and high expression of N-cadherin was increased in co-culture, compared to the uni-culture condition (Fig. 3C). These results indicated that the co-culture with HSC activated the Notch signaling pathway and induced EMT in HCC cells. In these experiments, both direct and indirect co-culture condition demonstrated similar results, indicating that the Notch signal activation and EMT were induced by soluble factors.

A number of soluble mitogens are secreted by HSCs (20). As TGF-β and HB-EGF are strong EMT inducers, we performed gene silencing experiments using siRNAs against them. These siRNA-treated HSCs were cultured with HCC, and their proliferation and migration abilities were assessed (Fig. 4). The siRNAs successfully silenced the expression of TGF-β and HB-EGF, resulting in decreased cell migration ability in the co-culture (Fig. 4B).

Flow cytometric analysis confirmed that the siRNA also reduced EMT in HCC cells under the co-culture with HSCs (Fig. 5). The results indicate that the interactions between HSCs and HCC cells were mainly induced by the soluble factors secreted by HSCs.

The effect of HSC co-culture was relatively weak in the immunoblot analysis, presumably as a result of the low concentration of the soluble factors. The Cell Culture Insert™ allowed us to assess the effect of soluble factors in the co-culture system while excluding the effect of cell-cell contact. The limitation of this co-culture system, however, is the relatively low concentration of soluble factors. The co-culture system required at least 3 ml of medium, which generates a low concentration of the soluble factors produced by 5×10^4^ HSC cells.

Another limitation of the present study is the use of immortalized HSCs. HSC cell line TWNT-1 may behave differently from natural HSCs, although TWNT-1 is known to have features similar to those exhibited by activated HSCs (25). This may be an advantage for elucidating the mechanism of HCC progression, since the active form of HSC contributes to it (21,26). Further studies are necessary to elucidate the role of HSC *in vivo*.

In advanced and metastatic HCC, the conventional treatment modality cannot achieve a curative effect. EMT and cancer stemness contributed to the poor prognosis in HCC (3–5,12,27). The activation of Notch signaling contributes to both EMT and stemness. Our results showed that HSCs activate the Notch signaling pathway and induce EMT, which indicates that HSCs may also activate CSCs. However, EpCAM-positive HCC cells were shown to have stem cell properties such as self-renewal, differentiation and tumor-initiating ability (6). It was also reported that higher EpCAM expression was related to the development of chemoresistance in HCC (28). Taken together, therefore, EpCAM has become recognized as a CSC marker in HCC (6,28). The interaction between HSCs and HCC cells may induce cancer progression by activating EMT and stemness. CSCs are resistant to the conventional therapeutic modality for HCC (29). A number of studies have demonstrated that high expression levels of CSC markers are related to the poor prognosis of HCC (3–5). Others have uncovered that EpCAM expression is also associated with poor prognosis (3–5). Thus, the interactions between HSC and HCC cells could be a therapeutic target for HCC. Further studies are necessary, however, to elucidate the effects induced by HSCs, particularly on CSCs.

In conclusion, we evaluated the effect of the cancer microenvironment on HCC by *in vitro* co-culture assay with HSCs. TGF-β and HB-EGF secreted by HSCs were shown to promote the acquirement of EMT and a stem cell phenotype by HCCs. TGF-β and HB-EGF could therefore be promising new therapeutic targets for CSC-based therapy.

## References

[1] European Association For The Study Of The Liver, European Organisation For Research And Treatment Of Cancer (2012). EASL-EORTC clinical practice guidelines: Management of hepatocellular carcinoma. J Hepatol.

[2] Venook AP, Papandreou C, Furuse J, de Guevara LL (2010). The incidence and epidemiology of hepatocellular carcinoma: A global and regional perspective. Oncologist.

[3] Bae JS, Noh SJ, Jang KY, Park HS, Chung MJ, Park CK, Moon WS (2012). Expression and role of epithelial cell adhesion molecule in dysplastic nodule and hepatocellular carcinoma. Int J Oncol.

[4] Schulze K, Gasch C, Staufer K, Nashan B, Lohse AW, Pantel K, Riethdorf S, Wege H (2013). Presence of EpCAM-positive circulating tumor cells as biomarker for systemic disease strongly correlates to survival in patients with hepatocellular carcinoma. Int J Cancer.

[5] Chan AW, Tong JH, Chan SL, Lai PB, To KF (2014). Expression of stemness markers (CD133 and EpCAM) in prognostication of hepatocellular carcinoma. Histopathology.

[6] Yamashita T, Ji J, Budhu A, Forgues M, Yang W, Wang HY, Jia H, Ye Q, Qin LX, Wauthier E (2009). EpCAM-positive hepatocellular carcinoma cells are tumor-initiating cells with stem/progenitor cell features. Gastroenterology.

[7] Bao B, Wang Z, Ali S, Kong D, Li Y, Ahmad A, Banerjee S, Azmi AS, Miele L, Sarkar FH (2011). Notch-1 induces epithelial-mesenchymal transition consistent with cancer stem cell phenotype in pancreatic cancer cells. Cancer Lett.

[8] Nishina S, Shiraha H, Nakanishi Y, Tanaka S, Matsubara M, Takaoka N, Uemura M, Horiguchi S, Kataoka J, Iwamuro M (2011). Restored expression of the tumor suppressor gene RUNX3 reduces cancer stem cells in hepatocellular carcinoma by suppressing Jagged1-Notch signaling. Oncol Rep.

[9] van der Gun BT, Melchers LJ, Ruiters MH, de Leij LF, McLaughlin PM, Rots MG (2010). EpCAM in carcinogenesis: The good, the bad or the ugly. Carcinogenesis.

[10] Patriarca C, Macchi RM, Marschner AK, Mellstedt H (2012). Epithelial cell adhesion molecule expression (CD326) in cancer: A short review. Cancer Treat Rev.

[11] Wu SD, Ma YS, Fang Y, Liu LL, Fu D, Shen XZ (2012). Role of the microenvironment in hepatocellular carcinoma development and progression. Cancer Treat Rev.

[12] Brabletz T (2012). To differentiate or not - routes towards metastasis. Nat Rev Cancer.

[13] Okamoto K, Tajima H, Nakanuma S, Sakai S, Makino I, Kinoshita J, Hayashi H, Nakamura K, Oyama K, Nakagawara H (2012). Angiotensin II enhances epithelial-to-mesenchymal transition through the interaction between activated hepatic stellate cells and the stromal cell-derived factor-1/CXCR4 axis in intra-hepatic cholangiocarcinoma. Int J Oncol.

[14] Kim SW, Choi HJ, Lee HJ, He J, Wu Q, Langley RR, Fidler IJ, Kim SJ (2014). Role of the endothelin axis in astrocyte- and endothelial cell-mediated chemoprotection of cancer cells. Neuro Oncol.

[15] Kikuta K, Masamune A, Watanabe T, Ariga H, Itoh H, Hamada S, Satoh K, Egawa S, Unno M, Shimosegawa T (2010). Pancreatic stellate cells promote epithelial-mesenchymal transition in pancreatic cancer cells. Biochem Biophys Res Commun.

[16] Hellerbrand C (2013). Hepatic stellate cells - the pericytes in the liver. Pflugers Arch.

[17] Osta WA, Chen Y, Mikhitarian K, Mitas M, Salem M, Hannun YA, Cole DJ, Gillanders WE (2004). EpCAM is overexpressed in breast cancer and is a potential target for breast cancer gene therapy. Cancer Res.

[18] Yang J, Mani SA, Donaher JL, Ramaswamy S, Itzykson RA, Come C, Savagner P, Gitelman I, Richardson A, Weinberg RA (2004). Twist, a master regulator of morphogenesis, plays an essential role in tumor metastasis. Cell.

[19] Coulouarn C, Corlu A, Glaise D, Guénon I, Thorgeirsson SS, Clément B (2012). Hepatocyte-stellate cell cross-talk in the liver engenders a permissive inflammatory microenvironment that drives progression in hepatocellular carcinoma. Cancer Res.

[20] Friedman SL (2008). Hepatic stellate cells: Protean, multifunctional, and enigmatic cells of the liver. Physiol Rev.

[21] Amann T, Bataille F, Spruss T, Mühlbauer M, Gäbele E, Schölmerich J, Kiefer P, Bosserhoff AK, Hellerbrand C (2009). Activated hepatic stellate cells promote tumorigenicity of hepatocellular carcinoma. Cancer Sci.

[22] Ju MJ, Qiu SJ, Fan J, Xiao YS, Gao Q, Zhou J, Li YW, Tang ZY (2009). Peritumoral activated hepatic stellate cells predict poor clinical outcome in hepatocellular carcinoma after curative resection. Am J Clin Pathol.

[23] Samatov TR, Tonevitsky AG, Schumacher U (2013). Epithelial-mesenchymal transition: Focus on metastatic cascade, alternative splicing, non-coding RNAs and modulating compounds. Mol Cancer.

[24] Tse JC, Kalluri R (2007). Mechanisms of metastasis: Epithelial-to-mesenchymal transition and contribution of tumor microenvironment. J Cell Biochem.

[25] Watanabe T, Shibata N, Westerman KA, Okitsu T, Allain JE, Sakaguchi M, Totsugawa T, Maruyama M, Matsumura T, Noguchi H (2003). Establishment of immortalized human hepatic stellate scavenger cells to develop bioartificial livers. Transplantation.

[26] Kang N, Gores GJ, Shah VH (2011). Hepatic stellate cells: Partners in crime for liver metastases?. Hepatology.

[27] Hashiguchi M, Ueno S, Sakoda M, Iino S, Hiwatashi K, Minami K, Ando K, Mataki Y, Maemura K, Shinchi H (2013). Clinical implication of ZEB-1 and E-cadherin expression in hepatocellular carcinoma (HCC). BMC Cancer.

[28] Kimura O, Kondo Y, Kogure T, Kakazu E, Ninomiya M, Iwata T, Morosawa T, Shimosegawa T (2014). Expression of EpCAM increases in the hepatitis B related and the treatment-resistant hepatocellular carcinoma. BioMed Res Int.

[29] Oishi N, Yamashita T, Kaneko S (2014). Molecular biology of liver cancer stem cells. Liver Cancer.

